# Condition of the Mouth in Idiots

**Published:** 1862-04

**Authors:** J. Langdon H. Down

**Affiliations:** London, Physician to the Asylum for Idiots, Earlswood


### ON THE CONDITION OF THE MOUTH IN IDIOTS.

By J. LANGDON H. DOWN, London,

*Physician to the Asylnm for Idiots, Earlswood.*

The condition of the idiot is not simply one of mental alienation. It frequently presents also grave physical deterioration; and this physical alteration is as much of idiocy as is the low condition of mental power. In a community such as that of the Earlswood Asylum there is to be found every variety of imbecile mind. In fact, just as in the outer world, there is a graduated series from the most commonplace intellects—who are 'the hewers of wood and drawers of water'—up to the giant minds that leave their impress upon the age in which they live; so is there among an imbecile population a gradual shading in an inverse direction—from the youth who might, if he had property, become the subject of inquiry before a master in lunacy, to one who, with every means of communication with the external world, except feeling, closed, vegetates in impenetrable mist. In such a community one can perceive the grades of physical condition accompanying the mental phases; and a study of the physical anomalies becomes as interesting and important as that of the psychological state. When contemplating so large a number as that which Earlswood shelters, one is able to set some of the members aside into natural groups, by simple reference to their physical state, and to predicate from that state what will be their probable future mental improvement. There is scarcely an organ in the body but may be found gravely altered in idiots; the circulation and respiration are abnormal; the skin exhibits perturbed functions; defective innervation, lesions of motility and nutrition, are abundantly met with; the bodily conformation is often of an aberrant kind. Regard, therefore, should be paid, in all cases of diagnosis of idocy, to the physical condition as confirmatory of any opinion based on purely psychological data. It is in this way one is enabled to differentiate an idiot from a simply backward or ill-regulated child. It is from the conviction of the importance of a study of the physiological manifestations of idiocy, that I have been induced to devote no small portion of time to an investigation into the structure and functions of the various organs *seriatim* among idiots and imbeciles. I purpose in the present paper giving some of the results of my observations of the feeble-minded, in reference to the condition and conformation of their mouths. Characteristic as is this region of various transitory mental phases among the sane, does it bear the permanent impress of a state in which the mind has failed in attaining its normal condition? If so, what is the nature of the impress? Does any value attach to the conformation of the mouth as confirmatory or otherwise of a state of mental incapacity? These are some of the questions we have to solve. I may premise that these observations have been made during the past year, without reference to any recent legal inquiry, and extend over 200 cases, which have been taken, without any special selection, from a large number. Not one on the list would, in the present condition, be able to manage his own affairs, or be legally held to be responsible. Many of them, however, are susceptible of considerable culture, are affected by the amenities of life, write letters to their friends, make small purchases, and form friendships. Several perform mechanical work with system and order. One, although possessing very little judgment, has been taught French and Latin, and reads these languages as well as ordinary schoolboys. Some few possess extraordinary memories and special aptitudes. One hundred and forty-six were males, and 54 females. Their ages ranged from 7 to 36.

*Palate.*—Among the 200 cases included in this inquiry, 82 possessed palates inordinately arched, and with this increased arching were noticed various abnormities. In some the palate was unsymmetrical, two sides having different degrees of concavity, or one side plane and the other concave. In 34 the palates were excessively arched, approximating to the appearance of the roof of a house, and with this extreme angularity was great narrowness. Excessive arching of the palate occurred, therefore, in 58 per cent. Excessive flattening of the palate was observed in 4 cases. In 34 cases, or 17 per cent., the palate had a very prominent antero-posterior ridge or keel corresponding to the line of approximation of the palatal bones. In 7 the plate bones did not meet, leaving a sulcus between them, the mucous membrane being, however, continuous. There was no instance of the ordinary cleft palate; and I may remark that, in an examination of nearly 600 idiots, I have failed in meeting with an example of that deformity. In several the hard palate extended but a short distance posteriorly, from defect of the palatal process of the superior maxillary bone and entire absence of the palatal process of the palate bone; and in all these cases the velum palati was unusually flaccid. In the majority of cases there was marked narrowness of the palate.

Thirty-three per cent. do not exceed 1 inch, and 62 per cent., while being more than 1 inch, do not exceed $1\frac{1}{4}$ inches; whereas the normal average has been stated to be $1\frac{1}{2}$ inches. It is worthy of notice that these numbers hold no direct relation to the age or stature of the patients examined. Thus, in a youth twenty-two years of age, and 6 feet 1 inch in height, so narrow is the palate that there is only 1 2·24th of an inch between the bicuspids, and only 10-24th of an inch between the opposite gums at their widest interval. The lowest measurements occurred in a boy and girl, the boy twelve, and the girl thirteen years of age. Neither is there a direct relation between the width of the palate and the cranial capacity; for in a microcephal whose palate was 22·24th of an inch wide, the internal canthi of the eyes were 23·24th of an inch distant from one another; while in a macrocephal whose palate was 22·24th of an inch wide, the distance between the internal canthi amounted to 2 inches.

*Teeth.*—The principal characteristics of the teeth in idiots are, that the period of the first dentition is delayed, the second dentition considerably postponed, and that they undergo very general and rapid decay. In many cases the anterior surface of the incisors presents a honeycombed appearance, but in no one instance have I observed those special characters which have been well shown by Mr. Hutchinson to be significant of congenital syphilis. In a large number of cases they are developed irregularly, are crowded, and the canine occupy a different plane from the other teeth—all these irregularities resulting from the imperfect development of the superior maxillary bone. In 6 cases, or 3 per cent., the upper incisors projected to such an extreme degree as to produce grave deformity. In 7 cases, the teeth of the lower jaw were in advance of those of the upper.

*Tongue.*—The most prevailing character noticeable in the tongue of idiots is the hypetrophy of the fungiform papillæ. Undue prominence of the papillæ was observed in 101 instances. In several there is a marked want of co-ordination in the movements of the tongue, so that the patient, although endeavoring to comply with the request, is unable to protrude it. This condition is usually associated with an absence of general co-ordinated movements, and in the improvement which is effected by treatment it is usually the most persistent derangement of motility. In 16 cases the tongue presented a soddened appearance, and exhibited deep, transverse furrows on its dorsal surface; in all these patients one is able to trace a marked physiological and psychological agreement, and so much do they resemble one another in these respects, that they might readily be taken for members of the same family. Inordinate size occurred in 12 instances, and in almost every case was associated with defective power of articulation. In 2 the tongue was unusually long; 33 were mute, 16 semimute. In 83 the speech was indistinct; in 62 the speech was fair. Stammering was observed in 4.

*Tonsils.*—One cause of the peculiar speech prevailing among idiots is the condition of the tonsils. These observations, for the most part, were made in the summer, when the tonsils were not likely to be rendered worse than their usual condition by climatic influences. In 30 instances they were injected; in 17 slightly enlarged, in 79 considerably enlarged; and in 5 so much increased in size as to interfere with deglutition and respiration.

*Mucous Membrane, etc.*—Besides the injection of the mucous membrane of the tonsils which has been noticed, other regions of the oral cavity are liable to this condition. The velum palati, uvula, and pillars of the pharynx were found to be thus characterized in 27 instances. The posterior wall of the pharynx was observed to be marked by considerable vascular injection in 33 cases, and in 6 the mucous membrane had assumed a granular appearance. The buccal and labial glands were generally hypertropoied, and the salivary glands were frequently enlarged. In 11 instances the sublingual gland was *greatly* enlarged. The uvala was elongated in 14 cases, bifid in 2, very short in 1, and entirely absent in 1. The lips were hypertrophied in 2. In 1 case only were the gums noticed to be swollen and tumid—a circumstance arising probably from the abundant supply of fresh vegetables with which the patients are provided.

*Slavering.*—The flow of saliva from the mouth is universally associated, in the popular mind, with the condition of idiocy. The slavering may vary in degree. It may occur only at periods of excitement, and at meal times, or with scarcely any intermission throughout the day, producing, in severe cases, excoriation of the chin. Among 325 cases which I have examined, I find 72, or 22 per cent., in which this habit was noticed. Of these, 28 slaver to a slight extent, 17 rather more so, and 26 in an aggravated degree. This peculiarity depends, I believe, on two or three causes—1st, the increased secretion of saliva; 2d, the deformed condition of the mouth; 3d, the want of co-ordinated movements in the muscles of the tongue; and 4th, the absence of tonicity in the labial muscles. Seeing that slavering exists in 22 per cent. of imbeciles, the question may arise—Is it confined to this section of the community? I am not prepared to say that it is never associated with mental vigor; but I believe that, excluding childhood, old age, disease of the mouth, and neural lesions, slavering is very rarely unconnected with mental imbecility. Moreover, I have examined, with reference to this question, 1000 persons who are doing the everyday work of the world, without meeting with a single example.

*Summary*—We have thus seen that idiocy is not simply a cerebral lesion ; that it carried with it marked physical deviations, of which I have shown conspicuous examples in the mouth ; narrowed, arched, and unsymmetrical palates ; tardily developed, irregular, and rapidly decaying teeth ; a hyperæmic condition of the mucous membrane and glands ; elongated uvulas and hypertrophied tonsils ; large, enervated, and rugous tongues, deficient in co-ordinated movements and in their special function ; saliva secreted ordinately, and retained incontinently. Such are some of the characteristics of a class in which mental vigor is an abeyance, which should be taken in connection with the psychological state in diagnosis, and inculcate the doctrine that the physical condition of these unfortunates should be specially sought to be ameliorated by an improvement of their physical condition.—*Abridged from Lancet.* (*Dublin Medical Press.*)

